# Adrenergic Modulation of Visually-Guided Behavior

**DOI:** 10.3389/fnsyn.2019.00009

**Published:** 2019-03-20

**Authors:** Mario Treviño, Ricardo Medina-Coss y León, Elí Lezama

**Affiliations:** Laboratorio de Plasticidad Cortical y Aprendizaje Perceptual, Instituto de Neurociencias, Universidad de Guadalajara, Guadalajara, México

**Keywords:** adrenergic receptors, norepinephrine, visual contrast discrimination, visual acuity, neuromodulation, divisive gain modulation, signal-to-noise ratio

## Abstract

Iontophoretic application of norepinephrine (NE) into the primary visual cortex (V1) *in vivo* reduces spontaneous and evoked activity, without changing the functional selectivity of cortical units. One possible consequence of this phenomenon is that adrenergic receptors (ARs) regulate the signal-to-noise ratio (SNR) of neural responses in this circuit. However, despite such strong inhibitory action of NE on neuronal firing patterns in V1, its specific action on visual behavior has not been studied. Furthermore, the majority of observations regarding cortical NE from *in vivo* recordings have been performed in anesthetized animals and have not been tested behaviorally. Here, we describe how micro-infusion of AR agonists/antagonists into mouse V1 influences visually-guided behavior at different contrasts and spatial frequencies. We found that cortical activation of α_1_- and β-AR produced a substantial reduction in visual discrimination performance at high contrasts and low spatial frequencies, consistent with a divisive effect. This reduction was reversible and was accompanied by a rise in escape latencies as well as an increase in the group averaged choice variance as a function of stimulus contrast. We conclude that pharmacological activation of cortical AR regulates visual perception and adaptive behavior through a divisive gain control of visual responses.

## Introduction

The locus coeruleus (LC) releases norepinephrine (NE) to the central nervous system, modulating the activity of entire brain areas (Berridge and Waterhouse, 2003; Aston-Jones and Cohen, 2005). NE produces a variety of cellular effects which depend on the diffusion dynamics of its volume release, the subtype of the adrenergic receptor (AR) that is activated, and the nature of the cellular and synaptic targets (Atzori et al., 2016; Salgado et al., 2016). NE interacts with three families of G Protein-coupled receptors: α_1_-AR (intermediate affinity), α_2_-AR (high affinity), and β-AR (low affinity; (Atzori et al., 2016). When activated, α_2_-AR decrease the intracellular concentrations of cyclic adenosine monophosphate (cAMP), whereas β-AR increase it. In contrast, α_1_-AR activate phospholipase C (PLC), triggering the synthesis of intracellular diacylglycerol and subsequent activation of serine-threonine protein kinase C (PKC) and phospholipid metabolism (Atzori et al., 2016; Salgado et al., 2016).

Activation of both α_2_-AR and α_1_-AR lead to an overall inhibitory drive in the cortex by decreasing the number of amino-propionic-acid-receptor (AMPA-R)- and N-methyl-D-aspartate-receptor (NMDA-R)-mediated currents. Additionally, α_2_-AR activation reduces hyperpolarization-activated cyclic nucleotide (HCN) currents and increases GABAergic transmission. β-AR activation increases calcium (Ca^2+^) and Ca^2+^-dependent-potassium currents and AMPA-R- and NMDA-R-mediated currents (Salgado et al., 2012; Treviño et al., 2012; Terakado, 2014; Atzori et al., 2016). Notably, despite the detailed knowledge about the actions of NE at the cellular level, it remains an open question of how NE acts at an intermediate network level involving the collective action of multiple cells. AR exist in pre- and postsynaptic membranes of a variety of cell classes, including pyramidal cells, GABAergic interneurons, neuroglia and astrocytes (Sato et al., 1989; Salgado et al., 2011, 2012, 2016; Terakado, 2014; Atzori et al., 2016). Hence, it is probably not surprising that the activation of AR does not converge onto a single unequivocal function.

In mouse primary visual cortex (V1), both incoming NE fibers and AR co-exist throughout all cortical layers. Experiments performed in this circuit confirm that NE modulates cellular excitability through α_1_-AR and β-AR, suppressing and enhancing excitatory synaptic currents, respectively (Ego-Stengel et al., 2002; Kobayashi, 2007; Salgado et al., 2012; Terakado, 2014), whereas α_2_-AR modulate inhibitory transmission (Salgado et al., 2011). Yet, it is still unknown how exactly NE recruits these receptor systems to produce a composite action (see *v.gr*.; Salgado et al., 2012). One proposal regarding the integrative action of NE is that it could improve sensory responses either by: (1) directly increasing the gain and signal-to-noise ratio (SNR) of visual responses (Kasamatsu and Heggelund, 1982; Waterhouse et al., 1990; Drouin et al., 2007); or (2) reducing the overall spontaneous activity of cortical circuits (also referred to as “internal noise”; Videen et al., 1984). Some studies confirm that NE can indeed produce a strong depression both in spontaneous and evoked activity, without changing the functional selectivity of cortical units (Olpe et al., 1980; Videen et al., 1984; Sato et al., 1989; Ego-Stengel et al., 2002). Additional experiments in auditory and prefrontal cortices reveal that AMPA-R-mediated glutamatergic transmission is reduced in the presence of NE with a net enhancement of inhibitory conductances into pyramidal neurons (Kobayashi et al., 2000; Salgado et al., 2012; Atzori et al., 2016). However, despite such strong inhibitory action of NE on neuronal firing patterns in V1 *in vivo* (Ego-Stengel et al., 2002), the potential implications in visual performance due to AR activation have not been studied. In addition, several predictions about the circuit actions of NE derived from *in vivo* recordings in anesthetized animals have not been tested behaviorally.

Using an automated two-alternative forced choice task that we recently developed and validated (Treviño et al., 2018), here we explored the effects of acute activation of cortical AR on the visual responses of adult mice at different contrasts and spatial frequencies. We found that micro-infused NE into V1, acting through α_1_- and β-AR, produced a substantial reduction in visual discrimination performance at high contrasts and low spatial frequencies. This reduction was reversible and consistent with a divisive effect on visual responses. Micro-infused AR agonists also increased the escape latencies and choice uncertainty of the mice. Therefore, acute AR activation regulates visual perception and adaptive behavior through a divisive gain control of visual responses in adult mice.

## Materials and Methods

### Animals

We used eight-week-old C57BL/6J male mice (18–28 g) housed in groups of 2–3 mice in standard polycarbonate cages (Alternative Design, USA; 29.2 × 18.4 × 12.7 cm) under conventional laboratory conditions, with food (Rodent Lab Chow 5001, Purina) and water *ad libitum*. The housing room operated in a regular 12:12 h. light/dark cycle (lights on from 8:00 a.m. to 8:00 p.m.) with constant room temperature (22°C ± 2°C) and humidity (55 ± 20%). The animals were trained and tested in the light phase of the day, between 8 a.m. and 2 p.m., from Monday to Friday, each session consisting of max. Seventy trials/day, lasting ~60–70 min. We conducted all the experiments following the Mexican animal welfare guidelines (SAGARPA, NOM-062-ZOO-1999), in line with the NIH’s Guide for the Care and Use of Laboratory Animals. The ethics committee of the “Instituto de Neurociencias” (Universidad de Guadalajara, México) approved the experimental protocols for the experiments performed during this investigation (ET062017-243 and ET062018-265).

### Behavioral Experiments

We trained and tested the mice with an automated two-alternative forced choice (2AFC) water maze (Treviño et al., 2018). The apparatus consists of a hexagonal swimming pool with an internal decision zone leading to three interior arms. Each experimental trial consisted in projecting a “positive” conditioned grating stimulus (CS^+^, 0.04 cycles/degree) in one randomly chosen arm, whereas the other two arms projected non-reinforced stimuli (CS^−^, 50% gray screens). The mice were released into the pool starting from one platform inside an arm (randomly chosen) and gradually learned to swim toward the CS^+^ (correct choice when entering the CS^+^-arm). When entering the correct arm, they could reach one of the two elevated platforms (located symmetrically to the left and right side of the projecting screen) and rest from swimming. Otherwise, by choosing the CS^−^ (incorrect choice), the mice had to continue swimming until they found one of the elevated platforms in the CS^+^ arm. Each session began by carefully placing a mouse onto one of the two elevated platforms (randomly chosen) from an arm projecting the CS^+^. From this moment on, the automatic system took charge of performing the subsequent training trials. We assessed discrimination performance by calculating the percent of correct choices/mouse and measured the escape latencies as the interval between trial start and time of mounting an escape platform. To encourage discrimination learning, we increased the cost of producing an error by repeating the training trial until the animal made a correct choice (max. of five error repetitions). We defined this set of swims, ranging from 1 to 6, as a “training unit” (Treviño et al., 2013). We finalized the training phase when the animals reached a stable discrimination accuracy of ≥90% over two consecutive days (Supplementary Figure S1A; Treviño et al., 2012). Next, we determined the grating-vs.-gray psychometric curves by using static sine wave gratings with a low spatial frequency of 0.04 cycles/degree with variable contrast in % contrast|_repetitions_: 5%|_20_, 12.5%|_15_, 25%|_12_, 37.5%|_9_, 100%|_8_. We also measured the grating-vs.-gray visual acuity thresholds by using static gratings with variable spatial frequencies at 100% contrast (in cycles/degree)|_repetitions_: 0.04|_10_, 0.18|_10_, 0.27|_14_, 0.50|_16_, 0.72|_16_. The category intervals to sample the contrasts and spatial frequencies were optimized using a logarithmic scale (Treviño et al., 2016). We report visual acuities in cycles per degree at 24 cm from the projecting monitors. We restricted the spatial frequencies to full cycles to eliminate gradients in average luminance between screens (Treviño et al., 2012, 2013). We adjusted the brightness of the monitors and background luminance of the room to conduct the experiments in photopic (230 lux ± 2.5 lux at 24 cm from the monitors; cone-dominated vision) or scotopic (≤5 average lux; rod-dominated vision) conditions (Supplementary Figure S1B). The experimenter was not visible to the mice during trials. At the end of each training session, the animals were carefully dried with a towel and placed back in their home-cages. We placed the testing apparatus inside a quiet laboratory room without windows and lit with diffusely reflected light. We conducted all experiments in silence, with mobile phones switched off and in the absence of perfumes.

We tested the mice at various contrasts and spatial-frequencies to measure their visual thresholds. The probability of producing a correct response as a function of contrast (or spatial frequency) corresponds to the psychometric curve. We extracted such curves from the averaged choices from each mouse by fitting the following logistic equation:

$$y(x)=\frac{L}{1+e^{-k[x-x_{0}]}}$$

where *y(x)* is the probability of producing a correct choice at *x* contrast (or spatial frequency), *L* is the curve’s maximum value, *e* is the natural logarithm base, *k* is the slope and *x*_0_ is the *x*-value of the sigmoid’s midpoint. We defined the visual thresholds as the value of the logistic curve fit at which the animal performed at 75% correct choices (Treviño et al., 2012, 2013). We compared the effects produced by the AR agonists/antagonists by using trapezoidal numerical integration to calculate the area under the psychometric curves. In addition, we predicted the effects of the AR agonists by transforming the shape of the psychometric curves obtained from control conditions (i.e., from un-injected mice). We did this by using the following equation:

$${y(x)|}_{\text{agonist}}=\frac{\left(L-d_{y}\right)}{1+e^{-k\left[(1+d_{x})\cdot x-x_{0}\right]}}$$

where *L*, *k* and *x*_0_ correspond to the optimized control parameters, and *d*_x_ and *d*_y_ represent modifiable input and output gain components, respectively. We derived four main models from this equation: (1) No gain modulation (Model 1: *d*_x_ = 0, *d*_y_ = 0; free parameters: *K* = 2); (2) Input gain modulation (Model 2: *d*_x_ = variable, *d*_y_ = 0; *K* = 3); (3) Output gain modulation (Model 3: *d*_x_ = 0, *d*_y_ = variable; *K* = 3); and (4) Input/Output gain modulation (Model 4: *d*_x_ = variable, *d*_y_ = variable; *K* = 4). We used the Akaike Information Criterion (AIC) to identify the best predictive model (Treviño, 2016). Briefly, the second order *AIC* (*AIC*_C_) compensates for sample size by increasing the relative penalty for fits with small data sets:

$$AIC_{C}=n\cdot ln\left(\frac{RSS}{n}\right)+2K+\frac{2K(K+1)}{\left(n-K-1\right)}$$

where *RSS* is the residual sum of squares (i.e., the sum of the squares of the residuals) between the transformed model (Equation 2) and the empirical data (micro-infused AR agonist), *n* is the number of mice, and *K* is the number of free parameters. Next, we ranked all models by taking the best approximation with the most negative *AIC*_C_ and calculated the Δ*AIC*_C_ as the difference between the best model and the *AIC*_C_ for each model (i.e., the best model has a Δ*AIC*_C_ of zero). Finally, to calculate the Akaike weights (*w*_i_), we took the relative likelihood of each prediction and divided it by the sum of these values across all models, as follows:

$$w_{i}=\frac{e^{-0.5\cdot \text{Δ}AIC_{C}}}{\sum_{r=1}^{R}e^{-0.5\cdot \text{Δ}AIC_{C,r}}}$$

Overall, these coefficients take into account how well each model fits the data (using the *RSS*), favoring descriptions with fewer free parameters, as it penalizes the number of fitted parameters (*K*).

Because different data distributions can lead to similar appearances when visualized through bar-plots (Treviño et al., 2016), we also quantified the frequency distributions of the escape latencies. For some comparisons, these distributions were re-scaled by using gain factors given by the ratio of mean escape latencies from relevant conditions. To simulate discriminative choices, we used a drift-diffusion model (DDM; Smith and Ratcliff, 2004) with: (i) variable input gain modulation of the drift rate; or (ii) variable starting points (10,000 trials; upper threshold of 0.35; lower threshold of 0; non-decision time of 0.20; variability in the non-decision time of 0.01; variability in drift rate across trials of 0.05; step of 0.0001; and 10,000 points for each cumulative probability distribution). All analysis algorithms were written in MATLAB R2014a (MathWorks, Inc., Natick, MA, USA). Visual stimuli were created and projected using the Psychophysics Toolbox extensions (PTB-3), as described previously (Treviño et al., 2018).

### Pharmacological Micro-infusions

We performed surgical procedures to implant the cannulae as previously described (Treviño et al., 2018). Briefly, we sedated the animals with a mixture of fentanyl (Fenodid, 0.15 mg/kg i.p.; Laboratorios Pisa), midazolam (Dormicum, 6 mg/kg i.p.; Laboratorios Pisa) and dexmedetomidine (Dexdomitor, 0.5 mg/kg i.p.; Orion Pharma). We protected their eyes with ophthalmic lubricant (Eyelube; Hydroxypropyl Methylcellulose; Optixcare) and subcutaneously injected small amounts of lidocaine (Piscaína 2%; Laboratorios Pisa) at the incision points. Next, we bilaterally implanted 30-gauge guide cannulae (made of stainless steel, BD PrecisionGlide™ Needles) targeting V1 (−4.29 mm AP, 2.75 mm ML, 0.6 mm DV from the dura; (Treviño et al., 2018). We inserted stainless steel obturators into the guide cannulae to prevent clogging. From this moment on, mice were housed individually to avoid them from removing the obturators from other mice. Mice were given at least 5 days of recovery after surgery. To micro-infuse the animals, we removed the caps and obturators to insert the injectors and used a home-made micro-infusion pump to inject 500 nl into each hemisphere of V1 (Supplementary Figure S2). We injected the following agonists by using amounts that took their receptor affinities into account (Atzori et al., 2016; Treviño et al., 2018): muscimol (GABA_A_ receptor agonist; 12.5 nmol; NE; 0–50 nmol), methoxamine (α_1_-AR agonist; 4.9 or 39.7 nmol), isoproterenol (β-AR agonist; 5.6 or 42.5 nmol). Additionally, we used the following adrenergic antagonists: prazosin (α_1_-AR antagonist; 5 nmol), yohimbine (α_2_-AR antagonist, 5 nmol), propranolol (β-AR antagonist, 5 nmol; Constantinople and Bruno, 2011; Polack et al., 2013). We also explored additional routes to activate AR, by using systemic intraperitoneal injections of the following agents: methoxamine (α_1_-AR agonist; 5 mg/kg i.p.), isoproterenol (β-AR agonist, 6 mg/kg i.p.), propranolol (β-AR antagonist, 10 mg/kg i.p.), and atomoxetine (NE reuptake inhibitor, 3 mg/kg and 10 mg/kg i.p.; (Treviño et al., 2012; Mizuyama et al., 2016; Pfeffer et al., 2018). All the drugs were bought from Sigma-Aldrich and freshly prepared before infusions, using 500 nl/hemisphere of NaCl 0.9% as the vehicle. The micro-infusion lasted a maximum of 10 min at a rate of 0.1 μl/min (1.67 nl/s). We removed the injectors 5 min after finishing the injections to allow the diffusion of the drugs. The behavioral experiments were initiated 10 min after cortical micro-infusions or 30 min after systemic injections. The animals showed no signs of discomfort during or after injections. We waited at least 3 days without infusions before performing additional pharmacological manipulations on the mice. The mice had to be pre-tested with muscimol to confirm their behavioral sensitivity to V1 inactivation in order to participate in additional micro-infusion experiments. These test injections were always followed by 1 day of wash-out and 1 day of baseline behavior before exploring the effects of additional agents. After finishing all behavioral experiments, we anesthetized, transcardially perfused and euthanized the mice with sodium pentobarbital (100–150 mg/kg i.p.; Pisabental; Laboratorios Pisa). We only included results from animals in which we confirmed, with conventional histological procedures, the location of the implanted cannulae against a reference atlas (Paxinos and Franklin, 2001). The psychometric plots that we illustrate throughout this manuscript correspond to the group averaged choices from all the mice that fulfilled each pharmacological condition.

### Statistical Analysis

We used multiple comparisons of the choices of relevant groups of mice at different contrasts or spatial frequencies with repeated measures analysis of variance (RM-ANOVA) tests. Optimized psychometric parameters (*L, k, x*_0_; Equation 1) from the logistic fits were compared using Kruskal-Wallis (KW) tests followed by Bonferroni’s *post hoc* tests (also multiple comparisons), and cumulative distributions with Kolmogorov-Smirnov (KS) tests. Our statistical analysis did not consider the repeated use of animals. All our results are described and illustrated as averages ± SEM. Significance was set at *P* < 0.05.

## Results

### Measuring Visual Performance With Pharmacological Access to Mouse Primary Visual Cortex

**Figure 1:**
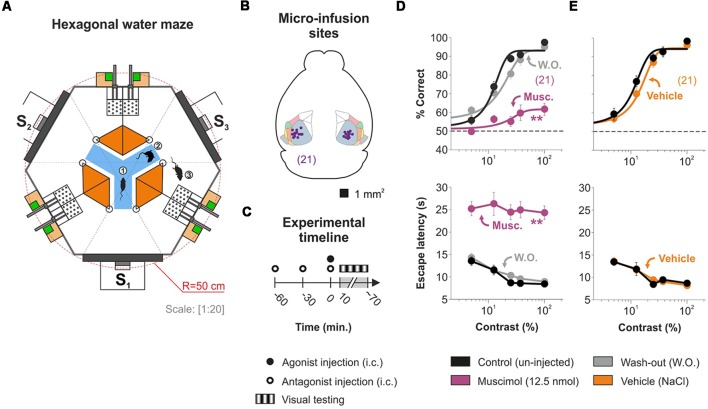
Characterizing mouse visual-cortex-dependent choices with the hexagonal water maze.** (A)** Schematic view of the hexagonal water maze. From the decision area (blue), the mice have visual access to one of three screens (S1–3). During each trial, a mouse can enter (1) and swim around (2) the decision area, and it can enter a chosen arm in search of an elevated platform (3). The presence of two equidistant platforms per arm has no impact on psychometric estimations (Treviño et al., 2018). **(B)** Implantation sites for 21 successfully cannulated mice. The micro-infused agents will directly affect the primary visual area (primary visual area (VISp, light blueish green), but they can also produce indirect effects *via*: (i) intra-module interactions with the lateral visual area (VISl, pale pink), rostrolateral visual area (VISrl, pink), posterolateral visual area (VISpl, sand), laterointermediate area (VISli, light yellow), anterolateral visual area (VISal, pale green), postrhinal area (VISpor, gray); and (ii) and inter-module interactions particularly with prefrontal cortex (Harris et al., 2018). **(C)** Timeline for the visual experiments involving micro-infusion of agonists, antagonists, or both. **(D)** Visual responses with variable contrasts of mice in control conditions (in black) are severely, but reversibly, impaired by the bilateral injection of 500 nl, 25 mM Muscimol (purple). **(E)** The micro-infusion of the same volume of saline solution (500 nl NaCl 0.9%, ~286 mM; orange) does not affect visual responses. Asterisks represent significant differences. Number of mice in parentheses.

We employed an automated water maze to study how the activation of AR located in V1 influenced the visual responses of adult mice (Figure 1A–C; Treviño et al., 2018). First, we trained bilaterally cannulated mice (Figure 1B) to discriminate a static stimulus (0.04 cycles/degree; 100% contrast) from 50% gray screens. All mice were behaviorally naïve to the task and began the first day of training producing correct choices at 50% chance level (Wilcoxon test, *P* ≥ 0.5). They quickly learned the task and reached high discrimination performance (≥90%) after 15 days of training (one-way ANOVA, *P* < 0.001, Bonferroni’s *post hoc* test, *P* < 0.001; *t*-test, *P* < 0.001). Accordingly, the escape latencies decreased asymptotically as learning progressed (one-way ANOVA, *F* = 13.72, *P* < 0.001, Bonferroni’s *post hoc* test, *P* < 0.001; *t*-test, *P* < 0.001). Next, by using equiluminant stimuli (Supplementary Figure S1B; Treviño et al., 2012, 2013), we characterized the visual discrimination performance of the mice, using either: (i) a low spatial frequency sine wave grating stimulus with variable permuted contrasts (i.e., to extract contrast psychometric curves); or (ii) a 100% contrast grating stimulus with variable permuted spatial frequencies (i.e., for visual acuity experiments). We then fitted logistic functions to the choice data to obtain a contrast threshold of 28.06% ± 2.28% (*n* = 21; Figure 1D) and a spatial resolution threshold of 0.50 ± 0.02 cycles/degree of visual angle (*n* = 7; Supplementary Figure S1D), consistent with previous observations from our laboratory (Treviño et al., 2012, 2013).

To confirm the effectiveness of our micro-infusion protocol (Figure 1C), we measured the effect of the GABA_A_ agonist muscimol on the psychometric curves (Figure 1D). Muscimol infusions into V1 produced a strong reduction of ~76% of the contrast responses (*n* = 21; % correct, *F* = 3.01, *P* < 0.001) and a ~80% drop in the visual acuity (reflected as a sliding to the left of the psychometric curve; *n* = 7; control: 0.50 cycles/degree ± 0.02 cycles/degree; muscimol: 0.07 cycles/degree ± 0.01 cycles/degree; Supplementary Figure S1D). This strong inactivation effect confirmed pharmacological access to V1, and thus, we consistently used it as an inclusion criterion to participate in the study (Supplementary Figure S3). Inactivation with muscimol was reversible 1 day after injection (*F* = 9.75, *P* < 0.001), and the micro-infusion of vehicle solution (NaCl 0.9%) alone did not affect visual function (*n* = 21; *F* = 0.51, *P* = 0.73; Figure 1E; Supplementary Figure S1E). This last result confirms that the micro-infused volume *per se* does not produce any change in the visual behavior of the mice. Averaged psychometric parameters and additional statistical comparisons are provided in Supplementary Table S1.

### Micro-infusion of NE Into V1 Reduces Visual Discrimination Performance in Adult Mice

**Figure 2:**
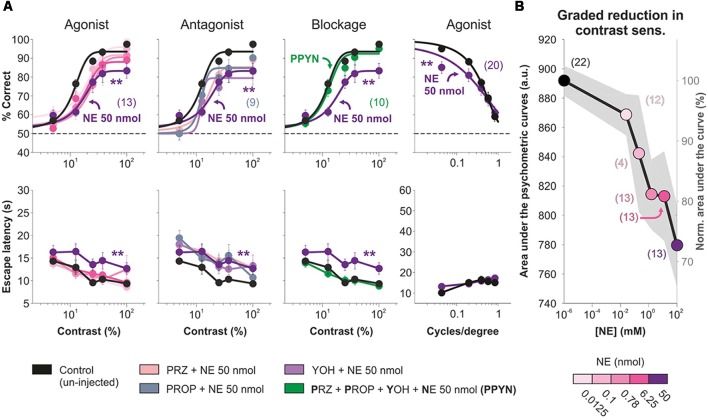
Primary visual cortex (V1) micro-infusion of norepinephrine (NE) reduces accuracy in visual responses of mice.** (A)** Micro-infusion of increasing amounts of NE (from 0 to 50 nmol) produces graded reductions in average visual contrast responses (panels on the left column) and average visual acuity (panels on the right column) of the mice. Pre-injection with the α_1_-adrenergic receptors (AR) antagonist prazosin (pink) or with the β-AR antagonist propranolol (desert blue), but not with the α_2_-AR antagonist yohimbine (violet blue), partially blocks the visual reduction by NE (panels on the second column). Full blockage of the effects produced by NE when pre-injecting the mice with a cocktail containing the three AR antagonists: prazosin + propranolol + yohimbine (green; panels on the third column). **(B)** Graded reduction in visual contrast responses with increasing concentrations of NE. Asterisks represent significant differences. Number of mice in parentheses.

Next, we activated cortical AR to explore their impact on visual performance. We bilaterally micro-infused 500 nl of different amounts of NE (0–50 nmol/hemisphere) into V1 and tested visual behavior 10 min afterward. NE produced a dose-dependent, reversible (not illustrated) drop in visual contrast performance reaching a ~27.59% reduction in the area under the psychometric curve (50 nmol NE, *n* = 13; *F* = 1.63, *P* = 0.04; Figure 2A) compared with control conditions (i.e., un-injected mice). NE did not affect the contrast required to produce half of the saturating response (i.e., contrast sensitivity; variable contrast | control: *x*_0_ = 86.76 ± 1.02, *n* = 22; NE, 50 nmol: *x*_0_ = 82.79 ± 2.96, *n* = 13; Multiple comparison KW test, *F* = 17.2, *P* < 0.01). This amount of NE also reduced the visual performance at different spatial frequencies by ~9.12% (area under the curve; *n* = 20, *F* = 2.42, *P* = 0.05; Figure 2A). To explore which AR could be mediating this effect, we injected mixtures of adrenergic antagonists before and together with the NE micro-infusion. The injection with 5 nmol yohimbine, an α_2_-AR antagonist, plus NE did not change the reduction observed with NE (*n* = 11; *P* = 0.6828) but was different to control conditions (*P* < 0.001). However, pre-injection with 5 nmol prazosin (α_1_-AR antagonist; *n* = 20) or 5 nmol propranolol (β-AR antagonist; *n* = 9) produced visual response curves that were similar to both control and NE conditions (*F* = 0.71, *P* = 0.1024), suggesting a partial blockage of the NE effect. Finally, a mixture of the three AR antagonists (prazosin + propranolol + yohimbine, 5 nmol each) rendered visual responses that were similar to control conditions but different to those observed with NE (*n* = 10; *F* = 0.90, *P* = 0.52; Figure 2A). Averaged psychometric parameters and additional statistical comparisons can be found in Supplementary Table S2. The overall reduction in visual discrimination performance by micro-infused NE can be appreciated when plotting the normalized area under the psychometric curves against agonist concentration (Figure 2B). Therefore, micro-infused NE caused a reversible reduction in visual discrimination performance which was produced mainly through α_1_-AR or β-AR, or both.

### Micro-infusion of α_1_- and β-AR Agonists Into V1 Reduces Visual Discrimination Performance in Adult Mice

**Figure 3:**
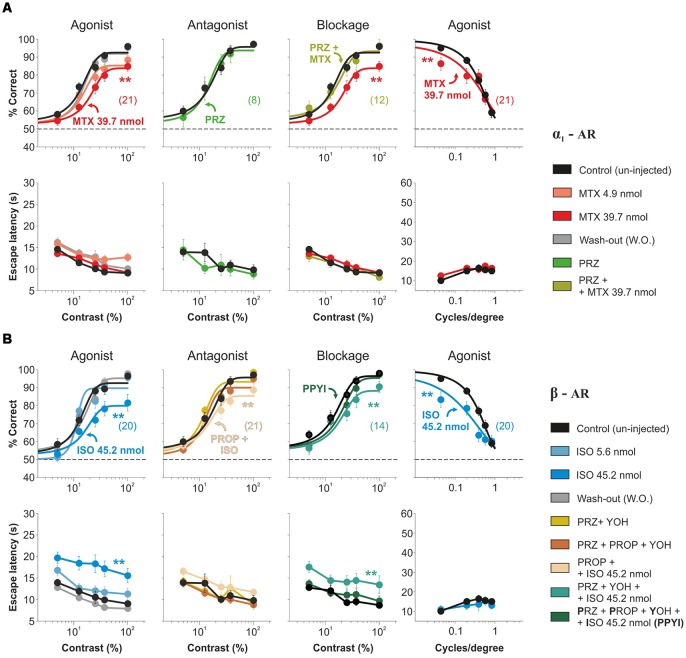
Visual impact of acute pharmacological manipulations of the α_1_- and β-AR systems in mouse V1.** (A)** Micro-infusion with the α_1_-AR agonist methoxamine (4.9 nmol, tropical pink and 39.7 nmol, red) into mouse V1 produces a reversible reduction in the accuracy of visual responses with variable contrasts (panels on the left column) and spatial frequencies (panels on the right column) of the mice. Pre-injection with the α_1_-AR antagonist prazosin alone (kelly green; panels on the second column) has no behavioral effect on itself, yet it blocks the reduction in visual contrast responses produced by the methoxamine (moss; panels on the third column). **(B)** Intracortical injection with the β-AR agonist isoproterenol (5.6 nmol, opaque sky blue, and 45.2 nmol, sky blue) produces a reversible reduction in the accuracy of visual responses with different contrasts (panels on the left column) and spatial frequencies (panels on the right column). These effects are partially blocked when pre-injecting the β-AR antagonist propranolol (sand; panels on the second column), but are blocked when including prazosin and yohimbine to the cocktail of antagonists (PPYI, Sacramento green; panels on the third column). Pre-injection with the antagonists alone (flax and brick red), has no effects on visual behavior (panels on the second column). Wash-Out (WO) traces in gray. Asterisks represent significant differences. Number of mice in parentheses.

We next aimed to isolate the contributions of α_1_-AR and β-AR on visual contrast responses. Micro-infusion of the α_1_-AR agonist methoxamine into V1 produced a reversible and dose-dependent reduction in visual performance at different contrasts (decrease of ~25.95% in the area under the psychometric curves with 39.7 nmol methoxamine/hemisphere; *n* = 21; *F* = 0.69, *P* = 0.76; Figure 3A). Although the micro-infusion of 5 nmol prazosin, an α_1_-AR antagonist, did not affect visual function (*n* = 8; *F* = 0.23, *P* = 0.92), a pre-injection with this antagonist blocked the methoxamine effects (*n* = 12; *F* = 0.58, *P* = 0.80; Figure 3A, Supplementary Table S3). Notably, the micro-infusion of isoproterenol, a β-AR agonist, also led to a reversible and dose-dependent reduction in the accuracy of visual responses at different contrasts (decrease of ~32.01% with 45.2 nmol isoproterenol/hemisphere; *n* = 20; *F* = 1.72, *P* = 0.06). However, the pre-injection of 5 nmol propranolol, a β-AR antagonist, did not block the isoproterenol effects (still with a remainder of ~17.76% reduction vs. control conditions; *n* = 21; *F* = 0.82, *P* = 0.63; Figure 3B). Because isoproterenol can activate α-AR at high doses (Bevan et al., 1977), we re-tested the effect of propranolol in the presence of a mixture of prazosin + yohimbine. In these conditions, propranolol fully blocked the isoproterenol effect (*n* = 11; *F* = 0.58, *P* = 0.79; Figure 3B, Supplementary Table S4). As we found for NE, separate activation of α_1_-AR and β-AR also reversibly reduced visual performance at different spatial frequencies (MTX: ~8.89% with 39.7 nmol methoxamine/hemisphere, *n* = 20; *F* = 3.06, *P* = 0.01; ISO: ~23.95% with 45.2 nmol isoproterenol/hemisphere, *n* = 20; *F* = 2.65, *P* = 0.03; panels on fourth column from Figures 3A,B). Interestingly, methoxamine, but not isoproterenol, decreased the contrast sensitivity by ~63% (MTX: *F* = 27.9, *P* < 0.001; ISO: *F* = 1.25, *P* = 0.74; Supplementary Tables S3, S4). These results indicate that acute activation of α_1_-AR and β-AR produced an overall decrease in visual performance at different contrasts and spatial frequencies.

### Adrenergic Receptors Produce a Divisive Control of Visual Responses in Adult Mice

**Figure 4:**
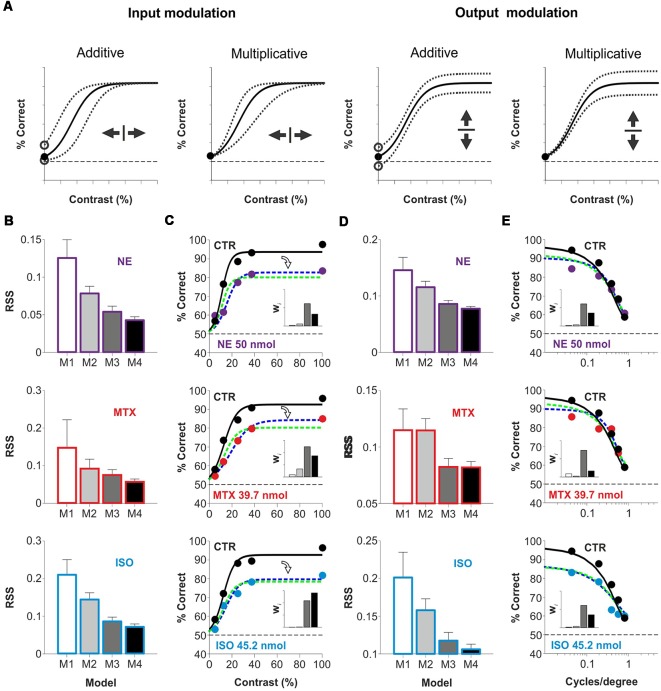
Acute divisive control of visual contrast responses by micro-infusion of adrenergic agonists in mouse V1.** (A)** The relationship between stimulus contrast (input) and visual discrimination choices (output) is represented with a contrast psychometric curve which is transformed by the action of neuromodulators. An additive transformation involves the sliding of the I/O curve along the x-axis (input modulation) or along the y-axis (output modulation). Similarly, a multiplicative transformation can produce a scaling of the I-O relationship along the x-axis (input modulation) or the y-axis (output modulation). However, only output gain modulation can scale the dynamic range of the output signal. **(B–E)** Comparison between four models (Model 1: white, Model 2: 30% black, Model 3: 70% black, Model 4: black; see “Materials and Methods” section) with variable I/O gain to describe the effects on the psychometric curves by NE (purple, superior row), methoxamine (red, midddle row) and isoproterenol (blue, inferior row). Panels (**B**; variable contrast) and (**D**; variable spatial frequency) show the residual sum of squares (RSS) values for Models 1–4 (see “Materials and Methods” section****; variable contrast, NE: *P* = 0.006, MTX: *P* = 0.16, ISO: *P* = 0.01; variable spatial frequency, NE: *P* = 0.03, MTX: *P* = 0.25, ISO: *P* = 0.16). Best psychometric fits (with smallest RSS) for Model 3 (green) and Model 4 (blue) appear in **(C,E)**, respectively. Inset bar-plots illustrate the Akaike weights for each model (see “Materials and Methods” section). These fits reveal that the actions of AR agonists on the psychometric curves can be described with a simple divisive output gain transformation.

The contrast psychometric function represents the relationship between stimulus contrast (input) and correct visual discrimination (output). Our results suggest that acute activation of cortical AR could transform the way V1 combines information influencing visual choices. Such transformation could act in an additive or subtractive fashion, regulating the number of driving inputs required for V1 neurons to reach their firing threshold. Alternatively, AR could also operate as a postsynaptic gain controller, to amplify signals or prevent their saturation, allowing efficient information transmission. There are multiple examples of multiplicative operations in a wide range of sensory systems and tasks (Silver, 2010; Carandini and Heeger, 2011; Katzner et al., 2011; Wilson et al., 2012). Because additive and multiplicative operations can occur at the input, the output, or both levels, we implemented a mathematical equation (Equation 2) to explore how AR transformed visual responses as a function of stimulus contrast/spatial frequency (Figure 4A). With the first model (Model I), we explored whether the psychometric curve observed with the agonist could be explained with no gain modulation of the psychometric curve observed in control conditions. The second (Model 2) and third (Model 3) models involved either input or output gain modulation, respectively, whereas the last model (Model 4), implemented a combination of both input and output gain modulation (see “Materials and Methods” section). We fitted the four models to the empirical data obtained with NE (50 nmol/hemisphere), methoxamine (39.7 nmol/hemisphere), and isoproterenol (45.2 nmol/hemisphere) and calculated their corresponding residual sum of squares (Figures 4B,D, Supplementary Figure S4, S5). Next, to identify the best description from the set of models tested, we used the second order (*AIC*_C_) and extracted the Akaike weights (*w*_i_) for each model (inset-bar plots in Figures 4C,E). Individual weights had a value between 0 and 1, corresponding to the probability that a given model constituted the best approximation (Σ*w* = 100%). Model 3 and Model 4 captured the strongest averaged weights for the three pharmacological conditions (M3|_contrast_: 51% ± 6%, M4|_contrast_: 41% ± 9%; M3|_spat.freq._: 65% ± 5%, M4|_spat.freq._: 29% ± 8%), indicating that they provided the best approximation. These analytic results reveal that micro-infused AR agonists into V1 produced a divisive control of visual responses in adult mice.

### Micro-infused AR Agonists Into V1 Increase the Escape Latencies of the Mice

**Figure 5:**
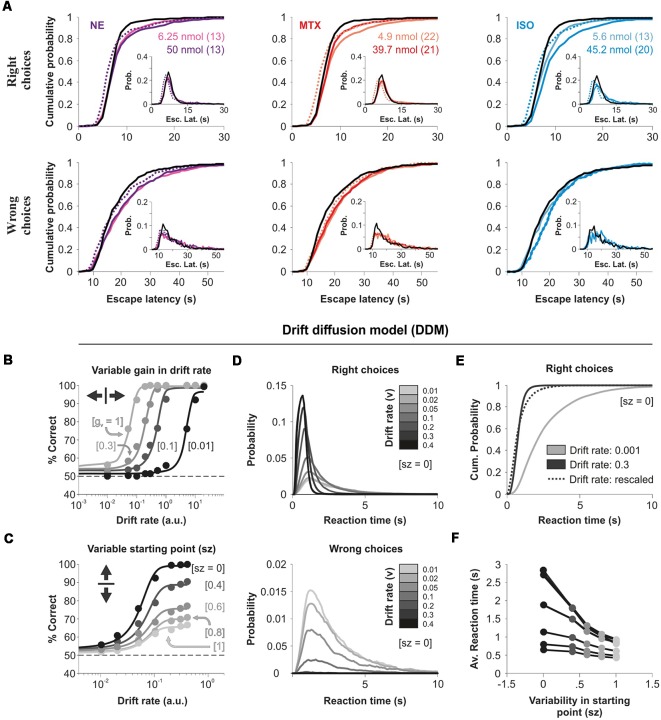
Micro-infused adrenergic agonists increase the escape latencies of the mice.** (A)** Cumulative probability distributions of the escape latencies for right (upper panels) and wrong (lower panels) choices for NE (left), methoxamine (middle), and isoproterenol (right) micro-infused mice. Insets show the relative frequency histograms. Dotted lines represent the scaled-up distributions of the latencies obtained with micro-infused agonists. **(B)** Simulation of choice accuracy with a drift-diffusion model (DDM) as a function of the drift rate (the model has variable drift gains, from left to right: 1, 0.3, 0.1, and 0.01). **(C)** DDM processes with variable starting points as a function of the drift rate (variable starting points, from top to bottom: 0, 0.4, 0.6, 0.8, 1). **(D)** Reaction time (RT) distributions for right (upper panel) and wrong (lower panel) choices with increasing drift rates (from gray to black) and a zero starting point. **(E)** No scalability between latency distributions obtained with different drift rates. **(F)** Smaller average RTs with higher variability in the starting point of the DDM. More details of the DDM in the “Materials and Methods” section.

Reaction time (RT) distributions constitute a rich source of information to understand perceptual processes. Factors such as stimulus saliency and the uncertainty of the responses influence the shape of these distributions (Treviño, 2016). Besides, there is ample evidence showing how the skewed shape of RT distributions depends on task difficulty and the rate at which information becomes available to solve it (Smith and Ratcliff, 2004). We, therefore, turned to distributional analysis to characterize the impact of the micro-infused agonists on the escape latencies of the mice. The escape latency distributions from all our experimental conditions were positively skewed (*S*_right_ = 13.00 ± 3.19, *S*_wrong_ = 5.75 ± 1.86, KW test, *P* = 0.0243), with the error responses being much slower than those for the correct responses (escape latency|_right_ = 9.19 s ± 0.30 s, escape latency|_wrong_ = 21.72 s ± 0.32 s; Figure 5A). Also, the infusion of the agonists produced an increase in the escape latencies involving relevant changes in the shape of the distributions because they could not be scaled-up to match the control ones (KS test, *P* < 0.01 for all cases; Figure 5A). Therefore, intra-cortical AR activation produced a non-multiplicative increase in the escape latencies for correct and wrong choices of the mice.

Because the DDM (Smith and Ratcliff, 2004) accounts for some empirical relationships found between correct and incorrect responses and their associated RT distributions, we wondered whether it could predict the reduced visual performance combined with the increased escape latencies of our mice. The DDM integrates discriminative information over time at a drift rate (*v*) that corresponds to the rate of accumulation of stimulus information: from a starting point (zero for non-biased systems) towards one of two response boundaries that trigger the response. We explored how the DDM predicted choices and RTs under a variety of testing conditions. An additive (not illustrated) or multiplicative control of the drift rate (*v*), produced a horizontal sliding of the input/output curve (Figure 5B). This transformation in the psychometric curve was orthogonal to the divisive changes that we observed in our experiments (compare panels from Figure 4C vs. Figure 5B). However, by randomizing the initial conditions of the DDM, we found that we could indeed increase the error rate and reduce the overall performance. This transformation on the output gain resembled our experimental results, but it led to a reduction in average RTs because trials lasted less when the initial condition started closer to the right/wrong boundary (Figures 5C,F). These simulations illustrate how the DDM could partially reproduce some properties of our escape latency distributions, but it could not predict a reduction in choice accuracy together with increased RTs. Consequently, we did not attempt to fit the DDM to our experimental data.

### Background Luminance Increases Choice Uncertainty Produced by Acute Activation of AR in V1

**Figure 6:**
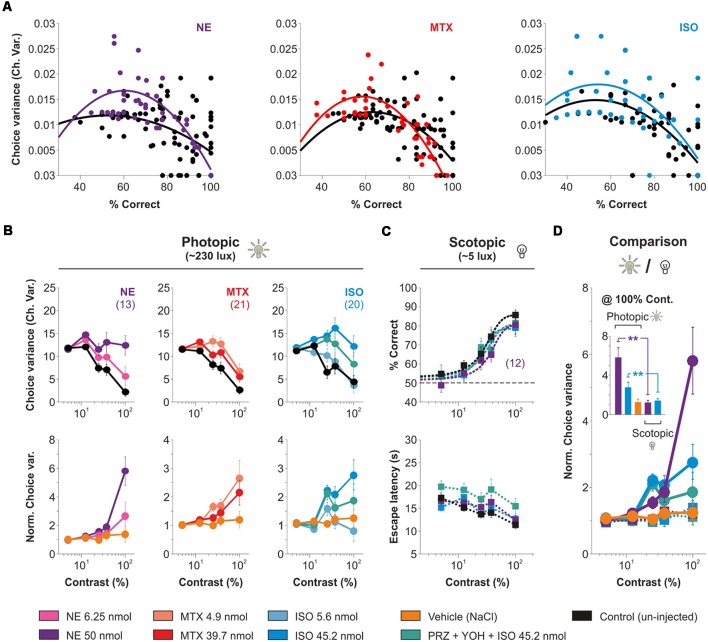
Contrast gradients in high background luminance increase the variability in visual behavior produced by the micro-infusion of adrenergic agonists.** (A)** Parabolic relationship between choice variance and average performance. Micro-infusion of AR agonists increased the choice variance compared to control conditions (same animals used for this comparison). **(B)** Experiments with a high background luminance of ~230 lux (i.e., photopic conditions; cone dominated vision). Upper panels depict the group average choice variance as a function of stimulus contrast with micro-infusions of (NE, 6.25 nmol, strong pink and 50 nmol, intense purple, left panels), methoxamine (MTX, 4.9 nmol, tropical pink and 39.7 nmol, red, middle panels), and isoproterenol (ISO, 5.6 nmol, opaque sky blue and 45.2 nmol, sky blue, right panels). Lower panels show normalized data against control conditions. **(C)** Mice can solve the task with a low background luminance of ~1–3 lux (i.e., scotopic conditions; rod-dominated vision). Micro-infusion with isoproterenol or NE produce no reduction in visual responses compared to control conditions (black). **(D)** Comparison of normalized choice variances in photopic vs. scotopic conditions. Note how the uncertainty produced by the AR-agonists increases with stimulus contrast and is most potent in high background luminance.

RTs tend to decrease with training because learning reduces signal and criterion uncertainty (i.e., reduced perception variance after training; Poort et al., 2015; Killeen et al., 2018). Furthermore, the uncertainty of making correct discriminations is inversely related to discriminability (d’), leading to lower RTs for correct compared to incorrect choices (Juslin and Olsson, 1997; Kiani and Shadlen, 2009). Therefore, we wondered whether the increased escape latencies observed with micro-infused AR agonists could be linked to an increase in the choice uncertainty of the mice. To explore this, we calculated the group mean average of the variance of correct choices extracted from individual mice from our experimental groups. The choice variance followed a parabolic relationship against the average performance (Figure 6A), confirming that changes in task performance should affect, non-linearly, the choice uncertainty of the mice. Therefore, we next explored how the AR agonists influenced the choice uncertainty of the mice. We found that the mice displayed a monotonic drop in the choice variance with increasing stimulus contrasts in photopic conditions (230 lux ± 2.5 lux; upper panels in Figure 6B). We implemented a measure to compare the uncertainty in the task by dividing the mean choice variance observed with micro-infused AR agonists against control conditions (a within-subject comparison). We found that the AR agonists, but not the vehicle solution (NaCl, yellow trace), systematically increased the relative choice variance as a function of stimulus contrast (NE: *F* = 3.99, *P* < 0.001; MTX: *F* = 2.11, *P* < 0.05; ISO: *F* = 2.83, *P* < 0.001; lower panels in Figure 6B). Notably. we repeated this measurement with experiments performed in scotopic conditions (~5 average lux; Figure 6C), but found a negligible increase in choice uncertainty by the micro-infused AR agonists (*F* = 0.88, *P* = 0.56; Figures 6C,D and Supplementary Table S4). These results indicate that micro-infused AR agonists strongly increased choice uncertainty as a function of stimulus contrast with high background luminance.

### Lack of Improvement in Visual Performance by Systemic or Intracortical NE

**Figure 7:**
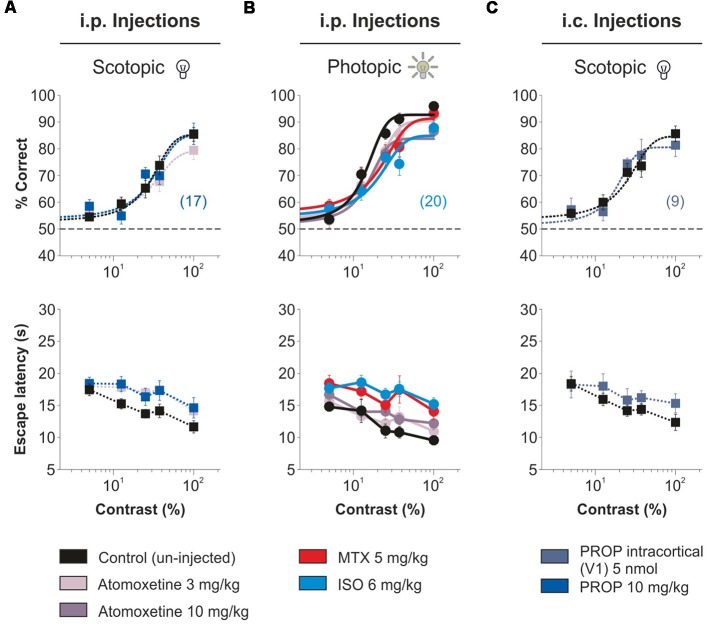
No improvement in visual contrast responses by adrenergic agonists/antagonists administered through different routes. Intraperitoneal injections with the NE reuptake inhibitor atomoxetine (two doses in different days: 3 mg/kg i.p., pale purple or 10 mg/kg i.p., grape), the β-AR antagonist propranolol (10 mg/kg i.p., persian blue), the α_1_-AR agonist, methoxamine (5 mg/kg, i.p., red) or the β-AR agonist, isoproterenol (6 mg/kg i.p., sky blue) 30 min. prior to visual tests do not improve visual responses in scotopic **(A)** and photopic **(B)** conditions. **(C)** Cortical micro-infusions of propranolol (5 nmol, blue sapphire) are also ineffective in increasing the accuracy of visual contrast responses.

A number of research groups propose that acute NE can increase the SNR in sensory systems (Videen et al., 1984; Waterhouse et al., 1990; Manella et al., 2017). For example, using systemic injections of propranolol, a β-AR blocker, a recent study suggests that endogenous NE could serve to “sustain” contrast sensitivity in young rats (Mizuyama et al., 2016). Another investigation reported that enhancing noradrenergic transmission with catecholamine reuptake blockers can boost sensory-evoked responses in anesthetized rats (Drouin et al., 2007). However, there are also many *in vivo* studies that indicate that NE can produce a generalized suppression of cell activity (Olpe et al., 1980; Sato et al., 1989; Kobayashi et al., 2000; Ego-Stengel et al., 2002). Given these apparent discrepancies, we wanted to confirm our main observations by using other means to activate AR. First, we tested the effects propranolol in the visual contrast responses evoked in low luminance conditions (Mizuyama et al., 2016). Neither systemic injections (“i.p. injections,” propranolol 10 mg/kg, *n* = 17; *F* = 1.15, *P* = 0.33; Figure 7A) nor intracortical micro-infusions (“i.c. injections,” propranolol 5 nmol, *n* = 9; *F* = 1.28, *P* = 0.28; Figure 7C) changed the contrast psychometric curves of the mice. Similarly, systemic injections of atomoxetine, an NE reuptake blocker, at concentrations of 3 and 10 mg/kg had no effect on the contrast responses of the mice both in scotopic (*n* = 20; *F* = 1.15, *P* = 0.33; Figure 7A) and photopic (*n* = 20; *F* = 1.54, *P* = 0.08; Figure 7B) conditions. Lastly, we explored the impact of using systemic injections of AR agonists, as we have done previously (Treviño et al., 2012). Neither methoxamine (*n* = 20) nor isoproterenol (~23.58% reduction; *n* = 20; *F* = 1.54, *P* = 0.08; Supplementary Table S5) increased the visual discrimination performance of the mice. A general conclusion from these experiments is that systemic and intra-cortical injections of AR agonists were ineffective in increasing the visual contrast responses of adult mice.

## Discussion

NE controls the dynamics of cortical networks enabling the transition across relevant behavioral states (Berridge and Waterhouse, 2003; Aston-Jones and Cohen, 2005). In V1, there is ample evidence of how NE modulates cellular excitability and synaptic responses through AR activation (Aston-Jones and Cohen, 2005; Salgado et al., 2012; Safaai et al., 2015; Atzori et al., 2016). However, the exact NE actions in visually-guided behavior remained unclear. Here, we investigated how micro-infusion of AR agonists into V1 interacted with sensory processing to modify visual behavior. Different routes of administration of AR agonists yield specific physiological responses due to the pharmacokinetics and distribution of receptors. Because systemic injections of AR agonists can yield strong peripheral effects, such as the activation of the cardiovascular system, we favored a method that allowed us to directly micro-infuse AR agonists/antagonists into mouse V1. The quick and localized effect of these intracortical injections precluded any direct action on peripheral receptors. The injected volumes diffused into the extracellular space of V1 and acted on AR expressed on the membranes of excitatory and inhibitory cells, all of them with diverse morphological and electrophysiological properties (Kobayashi et al., 2000; Salgado et al., 2011, 2012, 2016; Terakado, 2014). Our procedure involved injecting volumes of 500 nl of agonists which spread around the micro-infusion site (Allen et al., 2008), and acted on ~16–20% of the cell population in V1 (about ~46,000 pyramidal cells and ~6,000 interneurons per hemisphere; (Erö et al., 2018; Harris et al., 2018). Thus, our micro-infusions altered the function of a localized portion of the V1 microcircuit. Indeed, we found that NE micro-infusions produced a systematic and reproducible reduction in visual choices at high contrasts and low spatial frequencies. This observation is in agreement with other reports which indicate that NE can increase the frequency of spontaneous GABAergic transmission and the firing rate of interneurons *via* α_1_-AR while this, in turn, reduces spiking activity in pyramidal neurons suppressing top-down sources of information (Olpe et al., 1980; Sato et al., 1989; Ego-Stengel et al., 2002). In addition, we found that microinfusions of methoxamine and isoproterenol had strong inhibitory actions on visual responses. Previous reports indicate that activation of α_1_-AR reduces AMPA/NMDA currents in pyramidal cells in mouse V1, whereas β-AR increase excitatory and inhibitory responses in the cortex, with a substantial increase in GABAergic inhibition that reduces the spontaneous firing rate of the entire visual circuit (Olpe et al., 1980; Waterhouse et al., 1990; Salgado et al., 2012, 2016). Using competitive antagonists, we confirmed that the reduction in visual function by the micro-infused agonists was mediated through AR (Olpe et al., 1980; Sato et al., 1989). Interestingly, we found no changes in visual function when micro-infusing (or when using systemic injections) of the AR antagonists alone (but see; Sato et al., 1989; Mizuyama et al., 2016). Similarly, we found no acute effect in visual contrast responses when using systemic injections of atomoxetine, a selective NE reuptake inhibitor (but see Pfeffer et al., 2018). One possibility is that the high doses of agonists that we used during our first experiments caused degraded visual behavior. However, we reduced the amounts of all agonists by a factor of 1/4 and still found a suppressive action in visual responses. Furthermore, because isoproterenol could act on α_1_-AR due to their low affinity for this agonist (~1,000 μM for these receptors while for β-AR: ~80 nM), we conducted additional experiments injecting isoproterenol together with an α_1_-AR antagonist, yet we still found suppressive effects in these conditions.

One crucial feature we found is that AR activation reduced the gain of visual responses without affecting contrast sensitivity. Compatible with a divisive effect, AR can increase inhibitory conductances, scaling down EPSPs and reducing V1 activity (Silver, 2010; Wilson et al., 2012) without affecting the orientation tuning curves of V1 neurons (Ego-Stengel et al., 2002; Katzner et al., 2011; Wilson et al., 2012). Divisive modulation can explain how neuronal responses change with gratings of different sizes, contrasts, and orientations, such as why the spiking activity of cortical neurons saturates with increasing stimulus contrasts and why the cells fire fewer action potentials with bigger than with smaller stimuli (Carandini and Heeger, 2011; Wilson et al., 2012; Polack et al., 2013). Contrast discrimination is an essential sensory function that requires the observer to respond to one of two stimuli with a higher contrast. According to signal detection theory (SDT), performance on such a task depends on separate sensory and decisional processes. The sensory process depends directly on the physical properties of the stimuli, while the decisional process mediates the response. A general assumption in SDT is that the stimulus representation in the nervous system is noisy, and noise sources can be external (objective uncertainty) and internal (subjective uncertainty). Thus, to decide on a stimulus, the observer must accumulate sensory information by continuous sampling until the discriminative evidence for a response (signal distribution) surpasses an information criterion (C) that separates it from the noise level (noise distribution). In other words, the decision process requires comparing the perceived signal with an implicit decision criterion. If higher than the criterion, the stimulus is categorized as “signal” (S), otherwise as “noise” (N). After optimizing the criterion through experience (C → C*), an ideal observer will use the available information to detect the signal with maximum reliability, maximizing expected values (Killeen et al., 2018). From the SDT perspective, it is clear that manipulations of the signal or noise distributions, while keeping a stable criterion, should affect the discrimination process (Kiani and Shadlen, 2009). For example, manipulations that scale up or down both distributions will reduce the discrimination performance while keeping the SNR constant. Indeed, the only way to improve discrimination performance is to somehow exclusively reduce the noise distribution, or increase the signal distribution. Therefore, a simple explanation for our main results is that, by having a relatively similar action on cells from the local microcircuit, the injected AR agonists scaled the signal and noise distributions similarly, increasing the choice uncertainty and reducing visual discrimination performance of the mice.

Decision models predict an inverse relationship between RT and the strength of evidence (i.e., confidence; Baranski and Petrusic, 1994; Smith and Ratcliff, 2004). Moreover, a recent report suggests that catecholaminergic neuromodulators can increase the intrinsic variability of perception (i.e., “internal” uncertainty) and behavior, shifting the cortical computations underlying decision-making from stable to variable modes. More specifically, the pharmacological elevation of NE levels increases the variability in spike timings of cortical neurons and the rate of spontaneous perceptual alternations (Juslin and Olsson, 1997; Pfeffer et al., 2018). An additional study confirms this notion by showing how NE signaling within the cortex increases the variability in membrane potential of V1 neurons, thereby desynchronizing the circuit (Constantinople and Bruno, 2011). These reports and our observations that AR agonists increased the latencies and choice variance of the mice suggest that NE could act as an uncertainty signal.

For many years, we thought that V1 acted exclusively as a feature detector. However, recent *in vivo* evidence reveals that V1 neurons can also encode non-visual information. Training with predictive stimuli can change the responses and selectivity of V1 neurons. As mice learn to discriminate, V1 neurons become better at discriminating a rewarded from a non-rewarded stimulus, and less variable in their spiking activity (Poort et al., 2015). Behavioral factors such as arousal state, pupil dilation and the speed of locomotion modulate the gain and selectivity of V1 neurons. Both cholinergic and noradrenergic receptors have been linked to the locomotion-induced depolarization of V1 neurons (Constantinople and Bruno, 2011; Polack et al., 2013). The integration of visual flow with motor feedback is crucial to detect moving stimuli and to guide navigation. Furthermore, some V1 neurons can also encode the mismatch between the animal’s movement and the visual flow, consistent with a predictive coding strategy for visual processing (for a review, see Pakan et al., 2018). Thus, by modulating internal uncertainty, NE could influence the detection of discrepancies between the expected visual feedback (i.e., sensory predictions) and the actual visual input. Additionally, NE also plays a permissive role in gating experience-dependent plasticity in V1, indicating that AR can also alter sensory processing in longer time scales (Kobayashi, 2007; Salgado et al., 2012, 2016; Treviño et al., 2012; Atzori et al., 2016). Further studies to explore the impact of NE on experience-dependent plasticity should be of great significance.

One limitation of this work is that the behavioral task only allowed us to explore the impact of cortical micro-infusions in visual responses, but we did not record the spiking activity of V1 neurons and how it changed with the AR agonists. Moreover, given the particular experimental conditions we had, we could not extend the micro-infusion delivery to the whole testing period during which the mice solved the task. This is a well-known limitation of the technique and it implies that, through this study, we could not explore the *exact* role of phasic NE on the visual responses. However, despite the fact that AR activation produces acute *in vitro* effects that last for 10–15 min after washout (Salgado et al., 2012), many of the downstream actions on target proteins, like phosphorylation/de-phosphorylation of AMPARs, last for up to 2 h after treatment (Atzori et al., 2016; Salgado et al., 2016). This extended duration of intracellular effects makes the pharmacological micro-infusion of AR agonists into V1 a suitable approach to study the participation of these receptors on our visual task. Another consideration is that the particular distribution and orientation of pyramidal cells and their dendrites with respect to the injection cannulae could play an important factor in determining the circuit and behavioral responses that we characterized in this study.

In conclusion, this study explored the contribution of cortical NE to visual responses to stimuli with variable contrasts and spatial frequencies. The main result is that micro-infusion of α_1_- and β-AR agonists produced a divisive gain control of visual responses without changes in contrast sensitivity, consistent with the idea that these pharmacological manipulations have negligible effects on the orientation tuning of V1 units (Ego-Stengel et al., 2002; Katzner et al., 2011; Wilson et al., 2012). This work contributes to understanding how changes in the internal levels of NE can produce relevant changes in visual capacities.

## Author Contributions

MT conceived the project, designed and built all devices, created and adjusted the projection of visual stimuli, made analysis algorithms and analyzed data, made figures, drafted and wrote the manuscript. RM-CL performed surgeries, micro-infusions, histologies, adjusted the projection of visual stimuli, and helped make figures. EL performed behavioral experiments and histologies. All authors discussed and contributed to the final version of the manuscript. The order of author names corresponds to their relative contribution to this research and manuscript.

## Conflict of Interest Statement

The authors declare that the research was conducted in the absence of any commercial or financial relationships that could be construed as a potential conflict of interest.

## Supplementary Material

FIGURE S1 Visual training and testing of the mice. **(A)** An example of a learning curve from ten mice trained with the automated water maze. The first derivatives of the % correct responses (upper panel) or the “Escape latency” (lower panel) are illustrated as dotted lines and serve to identify when the dependent variables reach their asymptotic levels. Adjusted photopic luminances for the gratings with variable contrasts **(B)** and spatial frequencies (**C**; pool 1: light green, pool 2: light purple; similar calibration for stimuli in scotopic conditions, not illustrated). **(D)** Visual responses with variable spatial frequencies of sample mice in control conditions (in black) are severely, but reversibly, impaired by the bilateral injection of 500 nl, 25 mM Muscimol (purple). **(E)** The micro-infusion of the same volume of saline solution (500 nl NaCl 0.9%, ~286 mM; orange) does not affect visual responses. Asterisks represent significant differences. Number of mice in parentheses. The figures from **(A,D,E)** were adapted from Treviño et al. (2018).

FIGURE S2 Micro-injector pump. **(A)** 3D drawing of the micro-injector pump that we designed and built in our lab to micro-infuse small volumes (1–500 nl) of pharmacological agents at slow speeds of 0.1 μl/min directly into the mice’s brain. The system is based on controlling a stepper motor to an Arduino board mounted on a plastic chassis of 6 mm thick acrylic sheets. When triggered, a clockwise/anticlockwise step in the motor rotates the threaded bar which starts pushing/pulling forward/backward the plunger holder attached to an adjustable holder for two Hamilton syringes of 10 μl each (~1.67 nl/step). Polyethylene tubing connects the syringes with the infusion cannulae. **(B)** Orthogonal views of the same apparatus. For more details see Treviño et al. (2018).

FIGURE S3 Functional test to confirm pharmacological access to V1. **(A)** We used the profound visual impairment produced by the bilateral injection of 500 nl with 12.5 nmol muscimol into V1 (purple) as a simple test to confirm pharmacological access to this circuit (i.e., prior to pharmacological manipulations). **(B)** We excluded from the present study the animals that did not show these effects. Asterisks represent significant differences.

FIGURE S4 Gain control of visual contrast responses by micro-infusion of AR agonists in mouse V1. **(A)** Four models with variable input/output gain to describe the effects visual contrast responses by adrenergic agonists (see “Materials and Methods” section). Colored matrices show the residual sum of squares (RSS) with different combinations of I/O gain values (color-bars on the right). **(B)** Best fits obtained with Model 3 (green) and Model 4 (blue). Same representations and color coding for bar-plots as in Figure 4.

FIGURE S5 Gain control of visual acuity by micro-infusion of AR agonists in mouse V1. Four models with variable input/output gain to describe the effects on visual acuity by adrenergic agonists (see “Materials and Methods” section). Same representations, color coding, and figure legends as in Supplementary Figure S4.

TABLE S1 Micro-infusion of muscimol and vehicle solutions. Average psychometric parameters from the mice. This table corresponds to the experimental data we illustrate in Figures 1D,E. Arranged in columns: the experimental group, number of mice, the curve’s maximum value (*L*), the slope (*k*) and the sigmoid’s midpoint (*x*_0_). We used a multiple-comparison with a repeated-measures- (RM) ANOVA test to compare the choice data at different input contrasts/spatial frequencies, and a non-parametric Kruskal-Wallis test followed by a Bonferroni’s *post hoc* test to compare the optimized parameters from relevant experimental groups. Asterisks depict significant differences.

TABLE S2 Micro-infusion of NE. Same presentation of optimized parameters as in the previous table. The table corresponds to data we illustrate in Figure 2A.

TABLE S3 Micro-infusion of Methoxamine. Same presentation of optimized parameters as in the previous table. The table corresponds to data we illustrate in Figure 3A.

TABLE S4 Micro-infusion of Isoproterenol. Same presentation of optimized parameters as in the previous table. The table corresponds to data we illustrate in Figure 3B.

TABLE S5 Systemic vs. intracortical injections. Same presentation of optimized parameters as in the previous table. The table corresponds to data we illustrate in Figure 7.
