# Supplementary material for: Adrenergic Modulation of Visually-Guided Behavior
Source: Front Synaptic Neurosci. 2019 Mar 20;11:9. doi: 10.3389/fnsyn.2019.00009 (PMC6435528; doi:10.3389/fnsyn.2019.00009)
Supplement: TABLE S1 — Micro-infusion of muscimol and vehicle solutions. Average psychometric parameters from the mice. This table corresponds to the experimental data we illustrate in Figures 1D,E. Arranged in columns: the experimental group, number of mice, the curve’s maximum value (L), the slope (k) and the sigmoid’s midpoint (x0). We used a multiple-comparison with a repeated-measures- (RM) ANOVA test to compare the choice data at different input contrasts/spatial frequencies, and a non-parametric Kruskal-Wallis test followed by a Bonferroni’s post hoc test to compare the optimized parameters from relevant experimental groups. Asterisks depict significant differences. [file Data_Sheet_1.PDF]

Muscimol (12.5 nmol / hemisphere)

Variable Contrast	Mice	Multiple comparison of mean ranks: L	k	x_o	RM ANOVA
**Figure 1D (left upper panel)**		F = 34.78 P < 0.05 †	F = 4.23 P = 0.12 †	F = 1.38 P = 0.50 †	
Control	22	0.46 ± 0.01	0.76 ± 0.25	85.38 ± 1.40	F = 9.75
Muscimol	21	0.16 ± 0.03 *	1.28 ± 0.39	74.85 ± 6.45	P = 9.80 $^{-12}$
Wash-out	21	0.45 ± 0.01	0.22 ± 0.05	79.94 ± 2.88	

Variable Spatial Freq.	Mice	$L·10^{-4}$	k	x_o	
**Figure 1D (right upper panel)**		F = 1.47 P = 0.22 †	F = 6.86 P < 0.01 †	F = 1.80 P = 0.18 †	
Control	7	1.87 ± 1.00	0.18 ± 0.08	85.40 ± 36.95	F = 6.41
Muscimol	7	13.51 ± 5.53	3.04 ± 1.48 *	18.89 ± 10.95 *	P = 3.25 $^{-4}$

Vehicle (NaCl)

Variable Contrast	Mice	L	k	x_o	
**Figure 1E (left upper panel)**		F = 0.02 P = 0.88 †	F = 3.23 P = 0.07 †	F = 4.71 P = 0.06 †	
Control	21	0.45 ± 0.01	1.05 ± 0.32	88.03 ± 1.14	F = 0.51
Vehicle	12	0.46 ± 0.02	0.56 ± 0.28	84.23 ± 1.44	P = 0.73

Variable Spatial Freq.	Mice	$L·10^{-4}$	k	x_o	
**Figure 1E (right upper panel)**		F = 0.08 P = 0.78 †	F = 0.001 P = 1.00 †	F = 0.33 P = 0.57 †	
Control	7	1.87 ± 1.00	0.18 ± 0.08	85.40 ± 36.95	F = 0.93
Vehicle	6	2.03 ± 1.05	1.43 ± 0.94	109.16 ± 45.39	P = 0.45

† Kruskal-Wallis test w. Bonferroni *post-hoc* test

Supplementary table 1

NE

		Multiple comparison of mean ranks			RM ANOVA
Agonist (Var. Cont.) **Figure 2C (upper panel)**	Mice	*L* F = 13.9 P < 0.05 †	*k* F = 12 P < 0.05 †	x_o F = 17.2 P < 0.01 †	
Control	22	0.45 ± 0.01	0.51 ± 0.14	86.76 ± 1.02	*F* = 1.63
NE 0.0125 nmol	12	0.46 ± 0.02	0.51 ± 0.27	81.64 ± 2.78	*P* = 0.04
NE 0.1 nmol	4	0.44 ± 0.03	0.16 ± 0.08	77.92 ± 6.58	
NE 0.78 nmol	13	0.43 ± 0.02	0.37 ± 0.17	73.03 ± 4.18	
NE 6.25 nmol	13	0.37 ± 0.03 *	1.81 ± 0.54	84.21 ± 2.22	
NE 50 nmol	13	0.35 ± 0.03 *	0.45 ± 0.21	82.79 ± 2.96	
Antagonist (Var. Cont.) **Figure 2C (upper panel)**	Mice	*L* F = 7.46 P = 0.11 †	*k* F = 3.11 P = 0.53 †	x_o F = 2.16 P = 0.70 †	
Control	19	0.45 ± 0.01	0.75 ± 0.26	83.31 ± 1.99	*F* = 1.47
PRZ + NE	20	0.41 ± 0.02	0.84 ± 0.28	75.52 ± 4.91	*P* = 0.10
PROP + NE	9	0.41 ± 0.04	0.40 ± 0.07	80.60 ± 4.83	
YOH + NE	11	0.35 ± 0.05	1.10 ± 0.45	84.45 ± 3.03	
NE 50 nmol	13	0.35 ± 0.03	0.45 ± 0.21	82.79 ± 2.96	
Blockage (Var. Cont.) **Figure 2C (upper panel)**	Mice	*L* F = 6.94 P < 0.05 †	*k* F = 5.68 P = 0.06 †	x_o F = 0.09 P = 0.64 †	
Control	12	0.44 ± 0.02	0.46 ± 0.18	79.34 ± 3.51	*F* = 0.90
NE 50 nmol	13	0.35 ± 0.03 *	0.45 ± 0.21	82.79 ± 2.96	*P* = 0.52
PPYN + NE	10	0.42 ± 0.02	1.45 ± 0.42	81.65 ± 6.46	
Agonist (Var. S. Freq.) **Figure 2C (upper panel)**	Mice	$Lx10^{-3}$ F = 1.84 P = 0.18 †	*k* F = 0.79 P = 0.38 †	x_o F = 8.23 P < 0.01 †	
Control	21	3.00 ± 2.12	0.19 ± 0.02	34.00 ± 11.50	*F* = 2.42
NE (50 nmol)	20	27.03 ± 8.36 *	0.69 ± 0.45	128.02 ± 35.30 *	*P* = 0.05

† Kruskal-Wallis test w. Bonferroni *post-hoc* test

MTX

Agonist (Var. Cont.) Figure 3A (left upper panel)	Mice	Multiple comparison of mean ranks: L (F = 48 P < 0.01 †)	k (F = 18.71 P < 0.01 †)	x_o (F = 27.9 P < 0.01 †)	RM ANOVA
Control	23	0.45 ± 0.01	0.50 ± 0.18	83.50 ± 1.99	F = 0.69
MTX (4.9 nmol)	22	0.17 ± 0.04 *	0.57 ± 0.25	37.49 ± 9.25 *	P = 0.76
MTX (39.7 nmol)	21	0.15 ± 0.04 *	0.36 ± 0.19 *	30.20 ± 8.93 *	
Wash-out (W.O.)	21	0.45 ± 0.01	0.81 ± 0.25	79.87 ± 4.39	
**Antagonist (Var. Cont.) Figure 3A (upper panel)**	**Mice**	**L (F = 0.67 P = 0.41 †)**	**k (F = 0.002 P = 0.96 †)**	**x_o (F = 0.07 P = 0.79 †)**	
Control	23	0.45 ± 0.01	0.50 ± 0.18	83.50 ± 1.99	F = 0.23
PRZ	8	0.46 ± 0.02	0.81 ± 0.45	82.34 ± 3.77	P = 0.92
**Blockage (Var. Cont.) Figure 3A (upper panel)**	**Mice**	**L (F = 31.1 P < 0.01 †)**	**k (F = 12.6 P < 0.01 †)**	**x_o (F = 0.58 P = 0.80 †)**	
Control	23	0.45 ± 0.01	0.50 ± 0.18	83.50 ± 1.99	F = 0.58
MTX (39.7 nmol)	21	0.15 ± 0.04 *	0.36 ± 0.19 *	30.20 ± 8.93 *	P = 0.80
PRZ+MTX	12	0.46 ± 0.02	0.41 ± 0.19	84.20 ± 2.86	
**Agonist (Var. S. Freq.) Figure 3A (right upper panel)**	**Mice**	**$Lx10^{-3}$ (F = 0.08 P = 0.77 †)**	**k (F = 0.17 P = 0.68 †)**	**x_o (F = 0.94 P = 0.33 †)**	
Control	21	3.00 ± 2.12	0.19 ± 0.02	34.00 ± 11.50	F = 3.06
MTX (39.7 nmol)	20	24.08 ± 11.68 *	1.18 ± 0.61	67.30 ± 24.52 *	P = 0.01

† Kruskal-Wallis test w. Bonferroni *post-hoc* test

Supplementary table 3

ISO

		Multiple comparison of mean ranks			RM ANOVA
Agonist (Var. Cont.)	Mice	L	k	x_o	
**Figure 3B (left upper panel)**		F = 16.3 P < 0.01 †	F = 4.25 P = 0.24 †	F = 1.25 P = 0.74 †	
Control	22	0.44 ± 0.01	1.11 ± 0.33	82.64 ± 4.24	F = 1.72
ISO (5.6 nmol)	13	0.43 ± 0.02	1.64 ± 0.41	85.62 ± 2.55	P = 0.06
ISO (45.2 nmol)	20	0.35 ± 0.03 *	1.23 ± 0.35	76.85 ± 5.29	
Wash-out (W.O.)	21	0.47 ± 0.01	0.84 ± 0.30	81.92 ± 2.39	
Antagonist (Var. Cont.)	Mice	L	k	x_o	
**Figure 3B (upper panel)**		F = 3.20 P = 0.36 †	F = 2.10 P = 0.55 †	F = 4.72 P = 0.19 †	
Control	12	0.44 ± 0.01	1.11 ± 0.33	82.64 ± 4.24	F = 0.82
PRZ+YOH	12	0.47 ± 0.01	0.54 ± 0.27	85.60 ± 1.75	P = 0.63
PRZ+PRO+YOH	21	0.39 ± 0.04	0.55 ± 0.18	66.27 ± 7.40	
PROP+ISO	21	0.37 ± 0.02 *	1.07 ± 0.34	77.82 ± 5.15	
Blockage (Var. Cont.)	Mice	L	k	x_o	
**Figure 3B (upper panel)**		F = 5.89 P = 0.05 †	F = 2.66 P = 0.26 †	F = 0.15 P = 0.93 †	
Control	12	0.47 ± 0.01	0.19 ± 0.03	84.65 ± 2.63	F = 0.58
PRZ+YOH+ISO	14	0.39 ± 0.03 *	1.49 ± 0.48	81.64 ± 3.65	P = 0.79
PRZ+PROP+YOH+ISO	11	0.45 ± 0.01	0.94 ± 0.37	81.98 ± 3.70	
Agonist (Var. S. Freq.)	Mice	$Lx10^{-3}$	k	x_o	
**Figure 3B (right upper panel)**		F = 5.76 P < 0.05 †	F = 0.93 P = 0.33 †	F = 3.53 P = 0.06 †	
Control	21	3.00 ± 2.12	0.19 ± 0.02	34.00 ± 11.50	F = 2.65
ISO (45.2 nmol)	20	38.65 ± 12.85 *	0.96 ± 0.46	105.19 ± 25.19	P = 0.03

† Kruskal-Wallis test w. Bonferroni *post-hoc* test

Supplementary table 4

Scotopic conditions (~5 lux)

Variable Contrast	Mice	Multiple comparison of mean ranks: *L*	*k*	x_o	RM ANOVA
**Figure 6B (upper panel)**		F = 1.06 P = 0.79 †	F = 1.12 P = 0.77 †	F = 0.75 P = 0.86 †	
Control	21	0.37 ± 0.03	0.68 ± 0.20	66.29 ± 4.82	*F* = 0.46
NE (50 nmol)	12	0.33 ± 0.05	1.02 ± 0.39	61.78 ± 7.72	*P* = 0.93
ISO (45.2 nmol)	9	0.35 ± 0.05	1.42 ± 0.50	54.21 ± 12.04	
PRZ+YOH+ISO	12	0.33 ± 0.04	0.88 ± 0.33	69.43 ± 5.53	

Systemic injection and intra-cortical micro-infusion

i.p. Injections (Scotopic)	Mice	*L*	*k*	x_o	RM ANOVA
**Figure 7A (upper panel)**		F = 0.27 P = 0.87 †	F = 1.83 P = 0.40 †	F = 0.23 P = 0.89 †	
Control	21	0.37 ± 0.03	0.68 ± 0.20	65.03 ± 4.94	*F* = 1.15
Atomoxetine (3 mg/kg)	20	0.35 ± 0.03	0.79 ± 0.29	58.52 ± 7.12	*P* = 0.33
PROP (10 mg/kg)	17	0.38 ± 0.03	0.34 ± 0.20	68.28 ± 4.85	
i.p. Injections (Photopic)	Mice	*L*	*k*	x_o	
**Figure 7B (upper panel)**		F = 7.10 P = 0.13 †	F = 2.96 P = 0.57 †	F = 2.41 P = 0.66 †	
Control	20	0.45 ± 0.01	0.75 ± 0.26	83.31 ± 1.99	*F* = 1.54
Atomoxetine (3 mg/kg)	20	0.42 ± 0.02	0.74 ± 0.29	79.40 ± 2.76	*P* = 0.08
Atomoxetine (10 mg/kg)	20	0.40 ± 0.03	1.35 ± 0.40	77.12 ± 4.87	
MTX (5 mg/kg)	20	0.44 ± 0.02	0.75 ± 0.26	73.00 ± 4.58	
ISO (6 mg/kg)	20	0.39 ± 0.02	1.09 ± 0.41	78.23 ± 8.66	
i.c. Injections	Mice	*L*	*k*	x_o	
**Figure 7C (upper panel)**		F = 1.33 P = 0.25 †	F = 0.06 P = 0.80 †	F = 2.16 P = 0.14 †	
Control	21	0.37 ± 0.03	0.68 ± 0.20	65.03 ± 4.94	*F* = 1.28
PROP V1 (5 nmol)	9	0.34 ± 0.03	0.66 ± 0.39	77.39 ± 3.93	*P* = 0.28

† Kruskal-Wallis test w. Bonferroni *post-hoc* test
